# Supplementary material for: Population response magnitude variation in inferotemporal cortex predicts image memorability
Source: eLife. 2019 Aug 29;8:e47596. doi: 10.7554/eLife.47596 (PMC6715346; doi:10.7554/eLife.47596)
Supplement: Figure 2—source data 1. — Neural data include the spike count responses of each unit, averaged across novel and familiar presentations. The human-based memorability scores for each image are also provided. [file elife-47596-fig2-data1.zip › Figure 2b - Source Data 1/Readme.rtf]

All files are MATLAB files.Figure 2b - Source Data 1.mat:SCmat: a 707 unit x 107 image matrix containing the spike count responses of each of 707 units to 107 images, averaged across novel and familiar presentations.MBmat: a 27 session * 107 image matrix containing the memorability scores for each of 107 images in each of 27 sessions.
