# Supplementary material for: Population response magnitude variation in inferotemporal cortex predicts image memorability
Source: eLife. 2019 Aug 29;8:e47596. doi: 10.7554/eLife.47596 (PMC6715346; doi:10.7554/eLife.47596)
Supplement: Figure 2—source data 2. — Behavioral data include monkey performance when images were presented as familiar. The human-based memorability scores for each image are also provided. Ethics: Animal experimentation: All procedures were performed in accordance with the guidelines of the University of Pennsylvania Institutional Animal Care and Use Committee under protocol 804222. [file elife-47596-fig2-data2.zip › Figure 2c - Source Data 1/Readme.rtf]

All files are MATLAB files.Figure 2c - Source Data 1.mat:BEHmat: a 27 session * 107 image matrix containing the behavioral outcomes for each of 107 images in each of 27 sessions, where 1 = correct and 0 = wrong.MBmat: a 27 session * 107 image matrix containing the memorability scores for each of 107 images in each of 27 sessions.
