# Supplementary material for: Population response magnitude variation in inferotemporal cortex predicts image memorability
Source: eLife. 2019 Aug 29;8:e47596. doi: 10.7554/eLife.47596 (PMC6715346; doi:10.7554/eLife.47596)
Supplement: Transparent reporting form [file elife-47596-transrepform.docx]

***eLife’s* transparent reporting form**

We encourage authors to provide detailed information *within their submission* to facilitate the interpretation and replication of experiments. Authors can upload supporting documentation to indicate the use of appropriate reporting guidelines for health-related research (see [EQUATOR Network](http://www.equator-network.org/%20)), life science research (see the [BioSharing Information Resource](https://biosharing.org/)), or the [ARRIVE guidelines](http://www.plosbiology.org/article/info:doi/10.1371/journal.pbio.1000412) for reporting work involving animal research. Where applicable, authors should refer to any relevant reporting standards documents in this form.

If you have any questions, please consult our Journal Policies and/or contact us: [editorial@elifesciences.org](mailto:editorial@elifesciences.org).

**Sample-size estimation**

- You should state whether an appropriate sample size was computed when the study was being designed
- You should state the statistical method of sample size computation and any required assumptions
- If no explicit power analysis was used, you should describe how you decided what sample (replicate) size (number) to use

Please outline where this information can be found within the submission (e.g., sections or figure legends), or explain why this information doesn’t apply to your submission:

No explicit power analysis was used. A description of sample size is included: The sample size (number of successful sessions recorded) was chosen to match our previous work (Meyer and Rust, 2018).

**Replicates**

- You should report how often each experiment was performed
- You should include a definition of biological versus technical replication
- The data obtained should be provided and sufficient information should be provided to indicate the number of independent biological and/or technical replicates
- If you encountered any outliers, you should describe how these were handled
- Criteria for exclusion/inclusion of data should be clearly stated
- High-throughput sequence data should be uploaded before submission, with a private link for reviewers provided (these are available from both GEO and ArrayExpress)

Please outline where this information can be found within the submission (e.g., sections or figure legends), or explain why this information doesn’t apply to your submission:

How often each experiment was performed: Recording sessions began after the monkeys were fully trained on the task and after the depth and extent of IT was mapped within the recording chamber. Combined recording and behavioral training sessions happened 4-5 times per week across a span of 5 weeks (monkey 1) and 4 weeks (monkey 2).

Replication: Per the standard of the field, our results highlight replication across two monkeys (Figure 2 – figure supplements 2 & 4).

We would not consider any of our data an “outlier”.

Criteria for inclusion / exclusion: Criteria for inclusion of individual recording sessions is described.

**Statistical reporting**

- Statistical analysis methods should be described and justified
- Raw data should be presented in figures whenever informative to do so (typically when N per group is less than 10)
- For each experiment, you should identify the statistical tests used, exact values of N, definitions of center, methods of multiple test correction, and dispersion and precision measures (e.g., mean, median, SD, SEM, confidence intervals; and, for the major substantive results, a measure of effect size (e.g., Pearson's r, Cohen's d)
- Report exact p-values wherever possible alongside the summary statistics and 95% confidence intervals. These should be reported for all key questions and not only when the p-value is less than 0.05.

Please outline where this information can be found within the submission (e.g., sections or figure legends), or explain why this information doesn’t apply to your submission:

Statistical analysis method:

1) Pearson correlation: is used as a measure of correlation strength between variables that take on continuous values (Fig 2b, Figure 2 – figure supplement 2; Figure 2 – figure supplement 3; Figure 3; Figure 3 – figure supplement 1).

2) Point bi-serial correlation: is used as a measure of the correlation strength between one continuous and one binary variable (Figure 2c, Figure 2 – figure supplement 4).

Both are clearly labeled in the Figure legends.

Raw data: our dataset is large and never < 10. Raw data are plotted when possible (Figure 2b; Figure 2 – figure supplement 2; Figure 2 – figure supplement 3b)

Exact values of N: Values of N (number of images and units) are reported throughout the paper, as specified in the text and figure legends.

Definitions of center: Response magnitude (L2 norm) is plotted in Figure 2b & Figure 2 – figure supplement 2. Mean firing rate responses are presented in Figure 2 – figure supplement 3b.

Methods of multiple test correction: multiple tests were not applied.

Dispersion and precision measures: mean and standard error across sessions were reported for behavioral performance (Figure 2c, Figure 2 – figure supplement 4) and were calculated in the standard way. Mean and 95% CIs were reported for neural network responses (Figure 3; Figure 3 – figure supplement 1) and were calculated as described in the Methods.

Effect size: Pearson’s r and the Point bi-serial correlation were used, as described above.

Exact p-values are reported throughout the paper.

(For large datasets, or papers with a very large number of statistical tests, you may upload a single table file with tests, Ns, etc., with reference to sections in the manuscript.)

**Group allocation**

- Indicate how samples were allocated into experimental groups (in the case of clinical studies, please specify allocation to treatment method); if randomization was used, please also state if restricted randomization was applied
- Indicate if masking was used during group allocation, data collection and/or data analysis

Please outline where this information can be found within the submission (e.g., sections or figure legends), or explain why this information doesn’t apply to your submission:

Our subjects were not organized into experimental groups. Our study is a comparison of the relationship between neural signals and behavioral performance; it was performed in two monkeys.

**Additional data files (“source data”)**

- We encourage you to upload relevant additional data files, such as numerical data that are represented as a graph in a figure, or as a summary table
- Where provided, these should be in the most useful format, and they can be uploaded as “Source data” files linked to a main figure or table
- Include model definition files including the full list of parameters used
- Include code used for data analysis (e.g., R, MatLab)
- Avoid stating that data files are “available upon request”

Please indicate the figures or tables for which source data files have been provided:

Monkey behavioral data, neural data and image memorability scores are attached as source data.
